# Action on Invasive Species: Control Strategies of *Parthenium hysterophorus* L. on Smallholder Farms in Kenya

**DOI:** 10.1007/s00267-021-01577-5

**Published:** 2021-12-14

**Authors:** Martin Paul Tabe Ojong, Miguel Alvarez, Hanna J. Ihli, Mathias Becker, Thomas Heckelei

**Affiliations:** 1 International Food Policy Research Institute (IFPRI), Cairo, Egypt; 2 Institute for Crop Science and Resource Conservation, University of Bonn, D-53115 Bonn, Germany; 3 Institute for Food and Resource Economics, University of Bonn, D-53115 Bonn, Germany

**Keywords:** Herbicides, Invasive alien species, Land-use change, Probit model, Weed control

## Abstract

*Parthenium hysterophorus* L. (Asteraceae) is an invasive alien weed with detrimental effects on agricultural production, biodiversity, human and animal health, threating rural livelihoods in Asia and Africa. The problem emerged recently in the Kenyan Rift Valley, where it began to affect the landholdings of both agro-pastoralists and crop farmers. These vulnerable smallholders depend heavily on natural resources for their livelihoods. In this study, we assessed the severity of parthenium invasion and farmers’ management responses using a sample of 530 agro-pastoralists in Baringo County, Kenya, in 2019. We hypothesise that the implementation of existing management strategies depends on the state of parthenium invasion and household socio-economic characteristics. The prevalence and severity of parthenium invasion differed greatly among field plots. To control weeds, farmers resort to either hand weeding, the use of synthetic herbicides, or intensive tillage, sometimes in combination with mulching. A multivariate probit regression model shows that households’ characteristics determine the type of control strategies used as well as their complementarity and substitutability. Hand weeding is the most common option, adopted by almost 40% of farmers. The use of agrochemicals or soil-based control strategies appears to be related to knowledge and information characteristics such as access to extension services, membership in organisations and the educational level of household heads. While hand weeding and the use of synthetic herbicides depict significant substitutability, the latter strategy is limited to a few larger farms with market-oriented production. As parthenium invasion continues, policies need to improve farmer awareness and access to knowledge to enable pro-poor and environmentally sustainable control of parthenium on smallholder farms.

## Introduction

Invasive alien plant species continue to wreak havoc on many smallholder farms in developing countries, threatening food security and livelihoods. They have detrimental effects on crop yields, pasture productivity and tree plantations, but can also negatively impact the biodiversity of the natural environment (Mack et al. 2000; Duguma et al. 2019). Also, invasive plants can have negative impacts on human and animal health (Pratt et al. 2017; Witt et al. 2018; Rai and Singh 2020). With progressive land-use change and agricultural intensification, coupled with physical infrastructure development, biological invasions have increased over the past decade (Pyšek et al. 2020), and are predicted to continue to increase by at least one-third over the next two decades (Seebens et al. 2020). In general, anthropogenic disturbances and land degradation are the main causes of invasive dispersal dynamics (Witt et al. 2018).

*Parthenium hysterophorus* L., native to South America, belongs to the family Asteraceae and is considered a noxious weed and one of the most invasive species in the world (Shabani et al. 2020). *P. hysterophorus* (further referred to as parthenium) was accidentally introduced into Kenya and Ethiopia in the early 1970s (Tamado and Milberg 2000), possibly with contaminated wheat seeds from Australia (Pratt et al. 2017). Through allelopathic effects, parthenium inhibits seed germination and growth of a variety of crops and pasture species. Depending on water availability, it may flower throughout the year, increasing its dispersal pressure (unpublished data). In addition, the plant provides shelter and fodder (nectar) for mosquitoes that transmit viral diseases (Agha et al. 2020). Given its bioclimatic preferences, large areas of East Africa are suitable for parthenium spread, and many of these overlap with areas suitable for maize production (McConnachie et al. 2011). Losses in maize production due to parthenium invasion in Africa have been estimated at 3.8–7.7 million USD per year (Pratt et al. 2017), while parthenium-related yield losses in sorghum in Ethiopia range from 40 to 97% (Tamado et al. 2002).

While various chemical, physical and cropping system, management practices have been recommended to control or reduce the incidence of parthenium in croplands (Shabbir 2014; Duguma et al. 2019), few attempts have been made to (1) define under which social-ecological conditions farmers can effectively use these recommended practices, and (2) understand the inherent constraints to their use (Adkins and Shabbir 2014). While much attention has been paid to the drivers of parthenium spread and the economic losses it inflicts on production, little is known about the decision-making process for adopting different management strategies in farming systems in rural Africa. This is particularly important in parts of the Kenyan Rift Valley, where the spread of parthenium is a very recent phenomenon (Wabuyele et al. 2014) and where smallholder farmers are highly vulnerable because their livelihoods depend heavily on the quality of natural resources (Greiner et al. 2013). We hypothesise that the implementation of control strategies depends on the perceived and actual state of parthenium invasion and the socio-economic characteristics of households. This paper, therefore, examines the relevance of parthenium infestation in Baringo County in Kenya to smallholder decision making based on their socio-economic context and exposure to parthenium invasion.

## Materials and Methods

### Description of the Study Site

This study was conducted in Baringo County, which is located in the Rift Valley of Kenya. It is characterised by both lowlands and highlands with varying altitude, precipitation and vegetation formations. The highlands, represented in our study by the Tugen hills, are formed on volcanic rocks at altitudes of 800–2000 m above the sea level. They are mainly used as rangelands. The lowlands are located 700 m above the sea level with loamy sedimentary soils and alluvial clay deposits from the lakes. The undulating landscape is used for both crop- and rangelands. The area, also known as the Marigat plains, is bordered on the west by the Tugen hills, on the east by the Laikipia Escarpment, and on the north by the Elgeyo Escarpment and the Lake Baringo catchment. Administratively, the Baringo county is made up of six sub-counties (Baringo South, Mogotio, Eldama Ravine, Baringo Central, Baringo North and Tiaty). These sub-counties are further divided into smaller administrative sub-units like wards, locations and villages.

The area has an annual precipitation of 1000–1500 mm in the highlands and 300–700 mm in the lowlands (Mbaabu et al. 2020), which follows a bimodal distribution pattern with peaks in April and in November. Temperatures range from 10 °C (283.15 K) in the highlands to 35 °C (308.15 K) in the Marigat plains. Based on the Köppen–Geiger climate classification, Baringo falls into the BSh zone, which is a hot, semi-arid savannah climate (Peel et al. 2007). In terms of vegetation, the Baringo county has a mix of croplands, shrublands and forests. The shrubland and forests are home to tree species such as *Prosopis* *juliflora*, *Vachellia tortilis* and other woody perennials. There also are grasslands, pastures and reeds where livestock graze. Given that households are gradually shifting from livestock keeping to crop farming, many different crops like maize, beans, vegetables and fruits such as water melon are cultivated. In these croplands, farmers generally use different cultivation methods ranging from crop rotation, shifting cultivation to mixed farming.

The area has historically been settled by the Ilchamus and the Tugens, with some Pokots migrating seasonally from the north. The Ilchamus are a maa-speaking group, while the Tugens are Kalenjin-speaking. The main livelihood strategy of all these groups is semi-nomadic livestock keeping, which is increasingly being supplemented or replaced by rainfed maize cultivation and by irrigated agriculture near the shores of the lake (Greiner et al. 2013; Tabe-Ojong et al. 2021). Fishing and apiculture are also gaining importance as economic activities. Around the Lake Bogoria National Reserve and in areas near Lake Baringo, tourism and biodiversity conservancies are common. There, charcoal production is gaining importance as another new livelihood strategy and as part of management initiatives to reduce the negative impacts of the spread of another invasive alien species, *Prosopis juliflora* (Mwangi and Swallow 2008; Alvarez et al. 2019; Tabe-Ojong et al. 2021). Parthenium was first reported in Kenya in 1973 in coffee plantations. Since then has spread to other parts of the country including the Baringo country where it can be seen along irrigation channels and ephemeral lands (Wabuyele et al. 2014).

### Farm Household Survey

This study relies on a farm household survey, which was carried out between July and August 2019 using a two-stage sampling technique. In the first stage, 35 villages were randomly selected from four wards in the Baringo County. The wards were purposely selected due to the rapid spread of the invasive parthenium coupled with the established *Prosopis juliflora* invasion (Alvarez et al. 2019). Villages were selected in collaboration with the Kenya National Bureau of Statistics using the probability proportional to size random sampling technique. Village-level household lists were prepared with authorities in all 35 sampled villages. From these lists, 15–16 households were randomly selected^1^ in each village, resulting in a total sample size of 530 households.

Households were interviewed using a structured questionnaire developed based on initial visits to the study area. The questionnaire was designed on the World Bank’s Survey Solutions platform and tested before implementation. To ensure that respondents were able to understand the questions, the questionnaire was translated into the local languages, i.e., Ilchamus and Tugen. Interviews were conducted with household heads in their local languages at a time convenient to the households. Each interview lasted 45–75 min and questionnaires were administered by a team of eight enumerators who had received prior training.

The survey collected information on (1) awareness of parthenium as an invasive species, (2) management options to control its spread, (3) socio-economic and demographic characteristics of households and (4) access to information, extension contacts and membership in social organisations. To assess knowledge and awareness of parthenium, we showed pictures of parthenium and asked farmers if they could identify the plant. After they identified the plant, we jointly visited field sites where farmers had reportedly seen parthenium in both rangelands and crop fields. The presence of and/or damage caused by parthenium was quantified using a scale from 0 to 4, representing the percentage of ground cover (0 = no parthenium; 4 = 75–100% ground cover) in their crop fields.

### Statistical Analysis

A combination of descriptive and econometric methods was used to analyse the collected data. Descriptive statistics were used to assess farmer awareness and knowledge of parthenium, as well as reported severity and extent of damage. Descriptive statistics were also used to identify the different control options used by farmers.

While most farmers use single parthenium control options, some farmers apply multiple strategies that may be interrelated. In this regard, modelling the use of single-control options separately may mask potential complementary or substitutive effects of the different options. Failure to account for such interactions may result in biased effects since both the number and type of options adopted may be path-dependent (Cowan and Gunby 1996) and their adoption may be influenced by the experience of prior use. In view of assessing determinants for adopting control strategies and to account for possible interactions and intercorrelations in the error terms, we applied a multivariate probit model (MVP), implemented in the statistical programme STATA, and using the ‘mvprobit’ command for the conditional recursive mixed process (Roodman 2011). A positive correlation between two control options implies that the two options are complementary, while a negative correlation implies substitutive effects. The MVP is represented as a set of binary dependent variables (*Y*_*i*_) as shown below:

1 $$Y_{im}^ \ast = {{{{{\boldsymbol{X}}}}}}_{{{{{{\boldsymbol{im}}}}}}}\beta _{{{{{\boldsymbol{m}}}}}} + \varepsilon _{im}$$

2 $$Y_{im} = \left\{ {\begin{array}{*{20}{c}} 1 & {{{\rm{if}}}\,Y_{im}^ \ast \, > \, 0} \\ 0 & {{\rm{otherwise}}} \end{array}} \right.$$

$$m = 1,2,3$$

where *V* represents the farmer, *m* denotes the management choices available, $Y_{im}^ \ast$ is a latent variable representing utility differences in using the different management options. It captures the unobserved preferences associated with the use of these options. *Y*_*im*_ denotes the various management options used to control parthenium. *Y*_*im*_ is assumed to be a linear combination of observed characteristics ***X***_*im*_ representing a vector of household and farm characteristics thought to influence the use of different management options. ***β***_*m*_ is the vector of parameters to be estimated, while *ε*_*im*_ is the stochastic error term.

If farmers’ adoption of a particular control option is independent of the use of another one, the error terms may be independent and identically distributed, implying we could employ univariate probit models using Eqs. (1) and (2). However, as highlighted above, the errors terms may be correlated and follow a multivariate normal distribution (MVN) with a conditional mean of zero and a normalised variance of 1, whose covariance matrix is represented as (*ε*_*i,jm*_~MVN(0, ∑)) with

3 $${\sum} {\left[ {\begin{array}{*{20}{c}} 1 & {\rho _{12}} & {\rho _{13}} & \ldots & {\rho _{1m}} \\ {\rho _{12}} & 1 & {\rho _{23}} & \ldots & {\rho _{2m}} \\ {\rho _{13}} & {\rho _{23}} & 1 & \ldots & {\rho _{3m}} \\ \vdots & \vdots & \vdots & 1 & \vdots \\ {\rho _{1m}} & {\rho _{2m}} & {\rho _{3m}} & \ldots & 1 \end{array}} \right]}$$

Off-diagonal elements in the covariance matrix, *ρ*_*im*_, capture unobserved correlations between the various parthenium control strategies. The error terms are reflective of the unobserved factors driving the choice of the management option.

As the evaluation of the maximum likelihood function of an MVN requires multi-dimensional integration, we used the Geweke–Hajivassiliou–Keane simulator for recursive conditioning, thus viewing and evaluating the MVN as sequentially conditioned univariate normal distributions (Cappellari and Jenkins 2003).

## Results

### Knowledge of Invasion and Management Strategies Applied

**Fig. 1:**
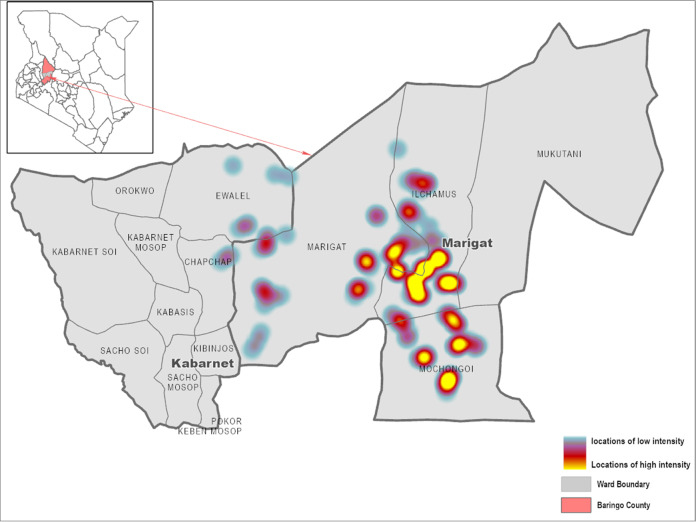
Study area showing reported intensity of the parthenium infestation and the share of ground cover in Baringo County in the northern region of Kenya. Marigat and Kabarnet (in bold) are the administrative headquarters of Baringo South and Baringo Central sub-counties, respectively. All other units represent some of the wards in the two sub-counties. Characterisation of parthenium intensity is based on farmer-reported levels of invasion, ranging from low (<25% infestation—blue colour), average (25–50% infestation—red colour), high (50–75% infestation—orange colour) to very high (>75% infestation—yellow colour). The percentages are the proportion of present/absent values in a range of fields. The map was created using ArcGIS mapping and analysis software

In terms of knowledge and awareness of parthenium, about 67% of the surveyed farmers correctly identified parthenium, with about 40% reporting invasions in their fields (Table 1). About 40% of the households had parthenium in both their pasture and croplands. Of these households, the ground cover of parthenium reported was about 65%. Knowledge of weeds and their harmful effects on crops or forage productivity is an important step in managing them. In most cases, knowledge of parthenium did not imply a direct infestation in individual farmers’ fields but could also concern invasion in neighbours’ fields. Figure 1 shows the spatial occurrence and reported extent of parthenium invasion in the study area.

**Table 1:** List of variables collected from a household survey on parthenium control strategies in Baringo County, Kenya

	Data type	Mean	SD
*Dependent variables (parthenium control strategies)*			
Manual weed removal in field crops	Binary	0.38	0.49
Spray application of herbicides	Binary	0.15	0.36
Intensive soil tillage	Binary	0.03	0.17
*Explanatory variables*			
Age of household head (years)	Continuous	45.15	15.62
Gender (male = 1, female = 0)	Binary	0.74	0.43
Education level (years of schooling)	Continuous	7.90	4.90
Cultivated area (ha)	Continuous	0.53	0.73
Household size (number of members)	Continuous	5.9	2.8
Level of parthenium invasion (%)	Categorical	0.65	0.99
Farming experience (years)	Continuous	13.4	13.0
Membership in producer organisation (Yes = 1, No = 0)	Binary	0.33	0.47
Access to credit (Yes = 1, No = 0)	Binary	0.43	0.50
Off-farm activities (Yes = 1, No = 0)	Binary	0.27	0.45
Extension contacts (Yes = 1, No = 0)	Binary	0.26	0.44
Use of M-pesa (Yes = 1, No = 0)	Binary	0.83	0.38
Livestock ownership (TLU^a^)	Continuous	3.2	5.1
Awareness of parthenium (Yes = 1, No = 0)	Binary	0.67	0.47
Infestation by parthenium (Yes = 1, No = 0)	Binary	0.40	0.49

M-pesa is a mobile money service

^a^TLU: tropical livestock units are units used to represent the different live weights of livestock in a bid to enable comparison of livestock ownership across households

Farmers in the study area used three main strategies to control parthenium, namely manual weeding, application of synthetic herbicides and intensive tillage, sometimes in combination with mulching. Hand weeding was the most popular management strategy. Nearly 60% of the surveyed households did not experience invasion of their plots with parthenium and hence did not apply any specific weed control strategy against parthenium. Among farmers affected by parthenium invasion, the largest share (90%), or 38% of all surveyed households manually removed parthenium from crop fields.

The second most popular option, used by 15% of all farmers (about 40% of those affected by parthenium invasion), was the application of synthetic herbicides. Only about 3% of households used intensive soil tillage such as ploughing, harrowing and mulching to control parthenium. While hand weeding is very labour-intensive, the use of synthetic herbicides requires financial investment to purchase agrochemicals, and the use of intensive tillage requires access to machinery or draft animals. These latter options were considered more desirable than hand weeding because they limit farmers’ physical contact with parthenium, which can cause allergic reactions in humans (Duguma et al. 2019). On the other hand, capital requirements limit the use of agrochemical and machinery-based options to better-off households with generally high wealth, with only about 3% of farmers in the sample meeting these conditions. Not a single farmer knew or used biological control measures, as recommended for example in South Asia (Adkins and Shabbir 2014).

### Socio-economic and Demographic Characteristics of Farmers

Farmers in the study area were on average 45 years old (Table 1), with slightly above a quarter of households being headed by women. The household heads had a moderate level of education of about 8 years of schooling, which is equivalent to primary plus post-primary school education. The households can be characterised as smallholders based on their farm size of about 0.53 hectares.

About one-third of the farmers in the sample belonged to associations such as cooperatives and saving groups. Off-farm income was quite low, with only about one-fourth of the households obtaining revenues from off-farm enterprises. Access to credit was available to just under half of the 530 households. Access to extension services was even lower, with only about 26% of farmers reporting contact with extension agents during the previous 12 months. In contrast, access to and use of a mobile money account was widespread, with about 83% of farmers reporting using M-pesa^2^ for financial transactions. Ownership of an M-pesa account requires ownership of a mobile phone, through which some farmers also reported to have received important extension information. Livestock ownership is represented by the Food and Agriculture Organization tropical livestock unit (TLU) index, where each livestock represents a specific unit, depending on its live weight. Since the study area is a pastoralist community, livestock ownership significantly represents rural wealth. Households reported owning an average 3.2 TLUs.

### Differential of Household Attributes based on Weed Management Strategies

We compared group means and standard deviations of key household attributes to understand socio-economic differences and drivers of adoption of different parthenium control strategies (Table 2). Younger household heads differed significantly from older ones in that the former used more herbicides, while the latter preferred manual weed control. Furthermore, male-headed households applied more herbicides than female-headed ones, suggesting gender-differentiated access to and use of external, capital- and knowledge-intensive inputs. Male-headed households are better connected with extension agents as a source of information and have more social capital (belonging to organisations), which may improve their access to knowledge and herbicides.

**Table 2:** Mean differences among households in Baringo, Kenya, based on the management strategies used to control *Parthenium hysterophorus*

Variables	Weeding		Herbicide application		Intensive cultivation	
	Yes (1)	No (0)	Yes (1)	No (0)	Yes (1)	No (0)
Age of household head (years)	40.740*** (0.954)	47.824 (0.892)	41.987** (1.389)	45.721 (0.758)	47.687 (3.431)	45.071 (0.691)
Household head is male (1 = Yes)	0.780 (0.029)	0.718 (0.024)	0.827** (0.042)	0.726 (0.021)	0.875 (0.085)	0.737 (0.019)
Educational level of household head	8.400* (0.327)	7.593 (0.274)	8.098 (0.541)	7.861 (0.229)	9.062 (1.062)	7.861 (0.215)
Area of cultivation (ha)	0.694*** (0.060)	0.422 (0.033)	0.886*** (0.135)	0.459 (0.027)	0.896* (0.216)	0.514 (0.031)
Household size (number)	6.515*** (0.188)	5.587 (0.150)	6.703*** (0.297)	5.799 (0.133)	5.812 (0.541)	5.941 (0.125)
Damage level (%)	1.655*** (0.065)	0.048 (0.015)	1.666*** (0.096)	0.472 (0.042)	1.312*** (0.150)	0.634 (0.043)
Experience in crop production (years)	11.335*** (0.712)	14.651 (0.792)	13.395 (1.132)	13.400 (0.636)	16.750 (3.939)	13.295 (0.570)
Farmer group (1 = Yes)	0.370 (0.034)	0.303 (0.025)	0.456*** (0.055)	0.305 (0.021)	0.500 (0.129)	0.322 (0.020)
Access to credit (1 = Yes)	0.565*** (0.035)	0.348 (0.026)	0.567*** (0.055)	0.405 (0.023)	0.322*** (0.020)	0.420 (0.021)
Off-farm participation (1 = Yes)	0.295 (0.032)	0.257 (0.024)	0.234 (0.047)	0.278 (0.021)	0.312 (0.119)	0.270 (0.019)
Access to extension services (1 = Yes)	11.335*** (0.712)	14.651 (0.792)	13.395 (1.132)	13.400 (0.636)	16.750 (3.939)	13.295 (0.570)
Use of M-pesa (1 = Yes)	0.920*** (0.019)	0.769 (0.023)	0.901** (0.033)	0.812 (0.018)	0.875 (0.085)	0.824 (0.016)
Livestock ownership (TLU)	3.895*** (0.368)	2.747 (0.271)	5.235*** (0.746)	2.810 (0.218)	4.429 (1.562)	3.142 (0.221)
Number of observations	200	330	81	449	16	514

Standard errors are shown in parentheses. The ‘1’ and ‘0’ in parenthesis signify dummies representing households’ use of particular strategies or not, respectively

***p < 0.01, **p < 0.05; *p < 0.1

Farmers with larger landholdings differed significantly from those owning little land, regarding the adoption of manual and chemical parthenium control strategies, while no apparent differences are observed regarding tillage-based weed control. Similar differences were also observed concerning household size (number of members in a household). There is a positive relationship between a large family and the availability of labour for manual weeding, a trend that is expected as manual weeding is usually labour-intensive and time-consuming. The trend appears less plausible for the observed correlation between household size and herbicide use. On the other hand, herbicide application is not scale neutral and may depend on a large family labour force for high levels of invasion, particularly in relation to large landholdings.

We also observed significant differences between households applying parthenium control strategies based on the extent of damage caused by parthenium. Farmers that never controlled parthenium had less crop damage. In addition, farmers who practised weed control were more experienced in growing crops and also had experienced parthenium infestations for longer times than farmers who did not weed. Their experience in managing other crop weeds probably increased their willingness (and the need) to manage parthenium. Farmers applying herbicides were usually members of village organisations, had larger landholding and more capital resources (owning portable phones and having larger numbers of livestock), and they had easier access to credit and extension support than their counterparts who are not members of such social groups.

### Covariate Relationship to Management Options

Because some of the covariates in the MVP model (Table 3) are potentially endogenous, we are not able to imply causal relationships and rather refer to these relationships as associations or correlations of farmers’ management options. Younger farmers are more likely to perform manual weeding than older ones. We also observed a positive correlation between the gender of the household head (male) and the use of manual weeding, though the variability in the sample of households was very large.

**Table 3:** Covariate relationships on farmer’s choice of parthenium management options in Baringo County, Kenya

	Weeding	Synthetic herbicides	Intensive cultivation
Age of household head (years)	−0.016** (0.009)	−0.009 (0.006)	0.012 (0.016)
Household head is male (1 = Yes)	0.092 (0.186)	0.244 (0.214)	0.225 (0.294)
Educational level of household head (years)	−0.006 (0.029)	−0.034** (0.016)	0.023 (0.028)
Area of cultivation (hectares)	−0.006 (0.075)	0.076*** (0.028)	0.031 (0.035)
Household size (number)	0.046** (0.023)	0.018 (0.020)	−0.039 (0.037)
Damage level (%)	2.750*** (0.461)	0.577*** (0.088)	0.254*** (0.078)
Experience in crop production (years)	−0.004 (0.006)	0.006 (0.007)	0.005 (0.013)
Farmer group (1 = Yes)	0.099 (0.125)	0.396*** (0.137)	0.163 (0.201)
Access to credit (1 = Yes)	0.195 (0.178)	−0.011 (0.190)	0.479* (0.274)
Off-farm participation (1 = Yes)	−0.022 (0.140)	−0.088 (0.158)	0.077 (0.240)
Access to extension services (%)	−0.014 (0.218)	0.426** (0.189)	0.068 (0.199)
Use of M-pesa (1 = Yes)	0.076 (0.197)	−0.133 (0.278)	−0.136 (0.357)
Livestock ownership (TLU)	−0.023 (0.034)	0.024** (0.012)	−0.004 (0.015)
Constant	−1.508** (0.742)	−1.635*** (0.384)	−3.210*** (1.202)
Ward dummies	Yes	Yes	Yes
Number of observations	530	530	530

Numbers in parenthesis present standard errors of the mean

***p < 0.01, **p < 0.05; *p < 0.1

While the educational level of the household head was not associated with the weed control strategies applied, the number of years in formal education has a negative relationship with the use of synthetic herbicides. Thus, each additional year spent in formal education reduced herbicide use by 3.4%. Finally, households cultivating larger areas of land are more likely to apply herbicides as a labour-saving strategy for parthenium control.

The perceived amount of damage caused by parthenium in farmers’ crop fields strongly enhanced the adoption of all three parthenium control strategies, and farmers who reported strong crop damage resulting from parthenium were more likely to apply any of the management strategies. Households belonging to village groups were more likely to use herbicides than households not belonging to such groups.

### Complementarities and Substitutability in the Management Options

Table 4 shows a significant correlation between the error terms of the use of weed control and the use of herbicides. The correlation is negative, indicating substitutability in the use of weed control and herbicide use. This means that farmers using synthetic herbicides are less likely to practice manual weeding. Since synthetic herbicide use is costly but less labour-intensive, this strategy dominates in large farms that have capital assets but have a shortage of labour. On the other hand, manual weeding is labour-intensive and time-consuming and hence dominates in smaller farms or in poor households with large numbers of members. No correlations were observed between the use of intensive tillage such as ploughing, harrowing and mulching and the use of manual weeding and/or the use of synthetic herbicides.

**Table 4:** Complementarity and substitutability of strategies to control the alien invasive weed *Parthenium hysterophorus* in Baringo County, Kenya

Strategies	Correlation coefficient
Synthetic herbicides versus weeding	−0.299** (0.130)
Intensive cultivation versus weeding	−0.115 (0.096)
Intensive cultivation versus synthetic herbicides	0.047 (0.139)

Numbers in parenthesis represent standard errors of the mean

**p < 0.05

## Discussion

### Management Options used by Smallholder Farmers

From the results presented, it is apparent that farmers in the Baringo County of Kenya apply different management strategies for the control of parthenium, including physical methods such as manual weeding, chemical control by herbicide application and cultural methods such as intensive soil tillage, sometimes in combination with mulching. While weeding is often described as an activity mainly carried out by women and children (Gianessi 2013; McConnachie et al. 2011), our analysis finds no evidence to support this claim. Weeding is also practised by male farmers. It is the strategy of choice for resource-poor households that rely mainly on family labour for all agricultural activities as financial constraints prevent the hiring of external labour or applying synthetic herbicides. The latter additionally requires access to knowledge as consistent dosing and timely applications are imperative to achieve good weed control (Goodall et al. 2010).

Some households resort to intensive soil tillage operations to control parthenium, both in the process of initial land preparation before seeding, but also by inter-row harrowing during early crop development stages. Such practices are time-consuming, they require access to rented tractors or own draft animals, and in the latter case as well as for manual weeding impact children who perform much of these activities. Thus, instead of going to school, children may spend considerable time during the early crop growth stages in weeding parthenium (Gianessi 2013; Pratt et al. 2017). Intensive tillage is costly, particularly when the renting of tractors is involved, requiring financial liquidity, for example through access to credit. Our analysis shows that households with access to credit are more likely to adopt intensive tillage, such as ploughing and mulching to control parthenium infestations.

One management option tested in mainly South Asia is biological control by spray application of microbial pathogens, such as *Puccinia abrupta* var. *partheniicola* (Dhileepan and Wilmot Senaratne 2009) or the release of insects that preferably feed on parthenium such as the leaf-feeding beetle *Zygogramma bicolorata* (Shrestha et al. 2015). While such biological control strategies, particularly when combining several control agents, have been successful and are widely advocated for parthenium control in South Asia, Australia and some parts of Africa, none of the farmers in Baringo was aware or used such approaches. This may be considered and possibly hinted at as a promising future control strategy of parthenium in the Central Rift Valley but would require support from both the public and possibly the private sector for establishing mass-rearing facilities in conjunction with the targeted release of these natural enemies. That said, non-usage by smallholder farmers in Baringo is not surprising as it requires breeding and releasing large number of control agents (insects mostly) that smallholders cannot afford (technically and financially). In Tanzania, *Zygogramma bicolorata* beetles have been released and found to be effective in managing parthenium both in the wet and dry season (Kanagwa et al. 2020).

### Covariate Association to Management Options

Farmers’ adoption of parthenium control strategies appears in the very first place to be driven by the intensity of invasion and the perceived extent of damage caused by parthenium to farmers’ fields. The MVP results showed that the choice of parthenium control strategies is correlated with several socio-economic factors that influence adoption. Among those attributes, farmers’ knowledge and access to information appear to be key to the adoption of options and effective control of parthenium invasions in pasture and croplands of Baringo. Farmers chose management options based on the level of invasion and their resource endowments. For example, resource-poor households with high labour availability, small landholdings and young household heads were more likely to choose manual weeding, while wealthier households with social ties and access to credit and extension services, as well as households with low labour input, were more likely to choose labour-saving mechanical (tillage-based) or chemical control strategies. Such relationships between farmers’ relative resource endowments and adoption of land-, labour-, capital- or knowledge-intensive crop management strategies were recently conceptually summarised by Becker and Angulo (2019) for systems in Asia. Accordingly, with small land areas and large numbers of members in the household, labour-intensive strategies such as manual tillage and hand weeding are favoured options. With access to machinery or draft animals, and particularly in larger landholding and labour-strapped households, intensive tillage appears to be the strategy of choice. Households practising large-scale cultivation are less likely to use weed control because it is more labour-intensive (Harrison et al. 2019), although the relationship is not statistically significant. Similarly, households with more household members are more likely to engage in manual weeding. Due to the lack of a market for labour in most rural areas, households rely largely on family labour for many agricultural activities such as weeding, which is both labour-intensive and time-consuming. Gitonga et al. (2010) and Tambo et al. (2020) obtained similar results in controlling *Liriomyza* spp. Leafminers and the fall armyworm in Kenya, Zimbabwe and Rwanda, respectively.

Households belonging to village groups were more likely to use herbicides than households not belonging to such groups. This result could be due to the fact that groups increase households’ access to farm inputs such as herbicides due to being able to purchase in bulk, making farmers obtain these herbicides at reduced and sometimes even subsidised prices. In summary, better-off or wealthier households are more likely to apply these agrochemicals. Thus, livestock ownership positively influenced herbicide use as livestock represents wealth in agro-pastoral societies.

Herbicide use is predominantly related to variables related to knowledge and information access such as household heads’ educational level, access to extension services, as well as wealth characteristics such as land and livestock ownership. This finding has implications for the design and formulation of invasive management strategies. Any intervention that improves the knowledge base and access to information for households would go a long way in strengthening farmers’ capacity to fight invasive species. Institutional characteristics such as access to credit also play a role in very costly management options such as the use of intensive farming methods. From an institutional perspective, our results suggest that households are constrained in accessing some services such as farmer groups and credit services. However, services such as the use of M-pesa are currently high. This also implies the prevalence of mobile phones and the associated benefits they offer.

### Substitutability between Hand Weeding and Herbicide Application

We also observed significant substitutability between the use of hand weeding and the use of herbicides, implying that any policy initiative that promotes the use of physical control methods such as weeding would greatly reduce the use of herbicides, which can be toxic to both humans and the environment. Furthermore, since weeding is labour- and energy-intensive (McConnachie et al. 2011; Gianessi 2013; Pratt et al. 2017), households may not fully utilise it. Since the substitution effect also works in the opposite direction, the use of synthetic herbicides may reduce dependence on weeding. Again, however, synthetic herbicides are costly and their successful application is both capital- and knowledge-intensive and will therefore be limited to wealthier households and farm types with market-oriented production. Improving access to herbicides (e.g., through subsidies) may reduce parthenium invasion and crop damage, but should be accompanied with adequate information on dosage and timing and on possible adverse health and environmental effects. Biocontrol strategies using natural enemies may be another viable alternative for future parthenium control in Baringo County and beyond. However, it should be noted that biocontrol agents may be more suited for pastoral lands and less suited in highly disturbed environments such as in crop production areas. In such areas, the use of an integrated management approach may be a win-win here (Adkins and Shabbir 2014; Lee and Thierfelder 2017).

## Conclusions and Outlook

Invasive alien species continue to cause detrimental effects on smallholder farms. This is particularly true for the invasive alien plant *P. hysterophorus* in Baringo County, Kenya, where the weed is spreading rapidly. While several management strategies have been proposed to control parthenium in pasture and croplands, it remains unclear whether farmers are adopting these options and if so, which option is most appropriate for which farm type or household category. This study addressed this knowledge gap through an on-farm survey involving 530 households. We show that depending on the intensity of parthenium invasion and the perceived damage it causes, the resource endowment of households, and particularly their access to knowledge are shaping adoption patterns.

The study has two limitations that should be addressed in future research. First, generalisations from Baringo County to other areas in the Central Rift Valley, to wider Kenya or rural Africa are limited due to the specific social-ecological context of the study area with specific biophysical features and the recent phenomenon of sedentarisation of former pastoralists. Second, we do not infer causal relationships and note that our analysis correlates potential determinants with the different management options used by farmers. As this is the first study of its kind on the emerging problem of parthenium spread in Kenya, future research can build on this work but needs to identify causal relationships in terms of targeting control options and upscaling recommendations.

## Footnotes

1 After constructing the household lists and identifying them with numbers, households were randomly selected using randomisation applications that randomly selects numbers.

2 M-pesa is a mobile phone-based money transfer service, payments and micro-financing service.
